# Proteomic Analysis of UV-B-Induced Virulence-Mutant Strains of *Puccinia striiformis* f. sp. *tritici* Based on iTRAQ Technology

**DOI:** 10.3389/fmicb.2020.542961

**Published:** 2020-10-02

**Authors:** Yaqiong Zhao, Pei Cheng, Yuzhu Zhang, Haiguang Wang

**Affiliations:** Department of Plant Pathology, College of Plant Protection, China Agricultural University, Beijing, China

**Keywords:** *Puccinia striiformis* f. sp. *tritici*, virulence variation, proteomics, iTRAQ, UV-B radiation, wheat stripe rust

## Abstract

The emergence of new physiological races of *Puccinia striiformis* f. sp. *tritici* (*Pst*) causing wheat stripe rust can lead to the loss of resistance of wheat cultivars to stripe rust, thus resulting in severe losses in wheat yield. In this study, after the germination of urediospores of three *Pst* strains including the original strain (CYR32, a dominant physiological race of *Pst* in China) and two virulence-mutant strains (CYR32-5 and CYR32-61) acquired from CYR32 via UV-B radiation, proteomic analysis based on isobaric tags for relative and absolute quantification (iTRAQ) technology was performed on the strains. A total of 2,271 proteins were identified, and 59, 74, and 64 differentially expressed proteins (DEPs) were acquired in CYR32-5 vs. CYR32, CYR32-61 vs. CYR32, and CYR32-61 vs. CYR32-5, respectively. The acquired DEPs were mainly involved in energy metabolism, carbon metabolism, and cellular substance synthesis. Furthermore, quantitative reverse transcription PCR assays were used to determine the relative expression of the 6, 7, and 1 DEPs of CYR32-5 vs. CYR32, CYR32-61 vs. CYR32, and CYR32-61 vs. CYR32-5, respectively, at the transcriptional level. The relative expression levels of one, five, and one gene, respectively, encoding the DEPs, were consistent with the corresponding protein abundance determined by iTRAQ technology. Compared with CYR32, the DEPs associated with energy metabolism and stress—including E3JWK6, F4S0Z3, and A8N2Q4—were up-regulated in the mutant strains. The results indicated that the virulence-mutant strains CYR32-5 and CYR32-61 had more tolerance to stress than the original strain CYR32. The results obtained in this study are of great significance for exploring the virulence variation mechanisms of *Pst*, monitoring the changes in *Pst* populations, breeding new disease-resistant wheat cultivars, and managing wheat stripe rust sustainably.

## Introduction

Stripe rust (yellow rust), caused by *Puccinia striiformis* f. sp. *tritici* (*Pst*), is a devastating wheat disease in wheat producing regions worldwide (Li and Zeng, 2002; Line, 2002; Chen, 2005; Wan et al., 2007; Chen et al., 2014; Wang et al., 2014). It can have a serious impact on wheat production, and can cause yield losses of 10–30% or even no yield once a disease outbreak or epidemic occurs (Li and Zeng, 2002; Wan et al., 2004, 2007). *Pst* can mutate in many ways to produce new strains or physiological races (Ma et al., 1993; Li and Zeng, 2002; Jin et al., 2010; Hu et al., 2014; Kang et al., 2015). The emergence of new physiological races can lead to the loss of stripe rust resistance in wheat cultivars and cause periodic epidemics of the disease (Li and Zeng, 2002; Chen, 2007; Chen et al., 2014; Hu et al., 2014; Kang et al., 2015). Hence, exploring the mechanisms of virulence variation of *Pst* can provide an important basis for wheat breeding and control of stripe rust.

Ultraviolet-B (UV-B) radiation (280–320 nm) is a part of sunlight, and can affect living things on Earth’s surface. In most reported studies, UV-B radiation has been used as a stress condition, and many aspects of its effects on living organisms have been investigated, including physiological and biochemical effects (Fargues et al., 1996; Jansen et al., 1998; Fernandes et al., 2007; Wargent and Jordan, 2013; Braga et al., 2015), genetic effects (Kumar et al., 2004; Braga et al., 2015), and effects on proteins (Wu X. C. et al., 2011; Trentin et al., 2015; Gao et al., 2019). As the main propagules of wheat stripe rust, *Pst* urediospores, in high-altitude areas of northwestern China with high-intensity UV-B radiation and in processes of long-distance dispersal with upper air flows, are affected by UV-B radiation, and virulence-mutant strains may be induced (Li and Zeng, 2002; Cheng et al., 2014; Hu et al., 2014; Kang et al., 2015).

Reported studies on the effects of UV-B radiation on *Pst* have focused on changes in the epidemiological components of *Pst* after UV-B radiation (Jing et al., 1993; Cheng et al., 2014), screening of virulence-mutant strains of *Pst* by UV-B radiation (Shang et al., 1994; Huang et al., 2005; Wang et al., 2009; Zhao et al., 2019), and random amplified polymorphic DNA (RAPD) analysis of the UV-B-induced virulence-mutant strains of *Pst* (Huang et al., 2005; Wang et al., 2009). An important study on *Pst* (Zheng et al., 2013) revealed the whole genome information of CYR32, a dominant physiological race of *Pst* in China, using high-throughput sequencing technology, which provided an important basis for the identification and functional verification of *Pst* proteins. More recently, a proteomics method based on isobaric tags for relative and absolute quantification (iTRAQ) technology, combining the isotope labeling method and tandem mass spectrometry, has been used to identify and quantify differentially expressed proteins (DEPs) between different proteomes, particularly in studies on plant-pathogen interactions (Fu et al., 2016; Xu et al., 2017; OuYang et al., 2018). Using this methodology, Zhao et al. (2016) investigated the difference between the proteomes of the urediospores of CYR32 before and after germination. The results showed that most of the DEPs were involved in biological processes such as carbon metabolism, energy metabolism, and transport. In our previous study (Zhao et al., 2018), the urediospores of three physiological races of *Pst* in China—CYR31, CYR32, and CYR33—were irradiated with a dose of UV-B radiation at which the relative lethal rate of the urediospores of each physiological race was 90%. Proteomic analysis of the irradiated urediospores, using methods based on iTRAQ technology, was then performed to explore the effects of UV-B radiation on *Pst* at the protein level. The results showed that most of the identified DEPs were mainly involved in energy metabolism, substance metabolism, and DNA biosynthesis. However, there are no reports on the differences in the level of protein expression between the original strains of *Pst* and the UV-B-induced virulence-mutant strains.

In our previous study (Zhao et al., 2019), two UV-B-induced virulence-mutant strains, CYR32-5 and CYR32-61, were screened from the UV-B-irradiated urediospores of CYR32 on the seedlings of the wheat cultivar Guinong 22. In this study, after germination of the urediospores of the original strain (CYR32) and the two UV-B-induced virulence-mutant strains (CYR32-5 and CYR32-61), proteins were extracted from the germinated urediospores and germ tubes, the DEPs among the proteomes of the three *Pst* strains were screened by using the proteomics method based on iTRAQ technology, and then the acquired DEPs were subjected to COG (Cluster of Orthologous Groups) annotations, KEGG (Kyoto Encyclopedia of Genes and Genomes) pathway analyses, and quantitative reverse transcription PCR (qRT-PCR) assays. This study is of great significance for exploring mechanisms of *Pst* virulence variation, and it can provide a reference for wheat resistance breeding and control of wheat stripe rust.

## Materials and Methods

### Materials

In the previous study (Zhao et al., 2019), the urediospores of the *Pst* physiological race CYR32 were irradiated with UV-B radiation under a radiation dose (the radiation intensity was 250 μw/cm^2^, and the radiation time was 95 min) for which the relative lethal rate of urediospores was approximately 90% (i.e., the relative germination rate of urediospores was approximately 10%); then the irradiated urediospores were inoculated on the seedlings of Guinong 22 to screen virulence-mutant strains. Finally, two virulence-mutant strains named CYR32-5 and CYR32-61, with stable infection types on Guinong 22 throughout four successive generations, were obtained. In this study, the original strain of the Chinese physiological race CYR32 of *Pst* and the two virulence-mutant strains (CYR32-5 and CYR32-61) were used. The wheat cultivar Mingxian 169, which is susceptible to all known Chinese physiological races of *Pst*, was used for *Pst* multiplication. Multiplication of the urediospores of each *Pst* strain was conducted in an artificial climate chamber (environmental parameters: light time, 12 h/d; light intensity, 10,000 lux; temperature, 11–13°C; and relative humidity, 60–70%) using the method described by Cheng et al. (2014).

### Germination of *Pst* Urediospores

Fresh urediospores of each of the three strains CYR32, CYR32-5, and CYR32-61 were collected from the diseased leaves of Mingxian 169 seedlings. For each strain, a spore suspension with a concentration of 2.5 mg/mL was prepared with 0.2% Tween-80 solution. Then, 4 mL of the spore suspension was transferred into a Petri dish (16 cm in diameter) containing 1% water agar medium. The urediospores (10 mg) contained in the suspension were evenly scattered in the Petri dish. When the suspension was almost dry, the Petri dish was sealed with plastic wrap and incubated for 10 h at 9°C in a dark environment. After incubation, a small agar block was picked up and placed on a glass slide for microscopic observation. At least 300 urediospores were checked, and the germination rate of the urediospores was recorded. A urediospore with a germ tube longer than half of the diameter of the urediospore was regarded as germinated. If the germination rate of the urediospores was more than 90%, the urediospores and germ tubes on the surface of the water agar medium were gently collected with a cover glass. For each *Pst* strain, the urediospores and germ tubes collected from five Petri dishes (approximately 50 mg in total) were treated as a sample.

### Protein Extraction and iTRAQ Labeling

In total, two samples of CYR32, three samples of CYR32-5, and three samples of CYR32-61 were acquired for protein extraction. Grinding tools were pre-cooled with liquid nitrogen, and then each of the prepared samples was pulverized with liquid nitrogen in a mortar and pestle. The pulverized sample was suspended in a trichloroacetic acid (TCA) and acetone solution (TCA:acetone = 1:10, w/v; pre-cooled at −20°C) and then precipitated for 2 h at −20°C. After centrifugation at 4°C under 20,000 × *g* for 30 min, the supernatant was discarded, and the precipitate was suspended in pre-cooled pure acetone, and then precipitated for 30 min at −20°C prior to centrifugation at 4°C under 20,000 × *g* for another 30 min. This process was repeated several times until the precipitate was substantially white. The precipitate was resuspended in a lysis buffer (8 M urea, 30 mM 4-(2-hydroxyethyl)-1-piperazineethanesulfonic acid, 1 mM phenylmethanesulfonyl fluoride, 2 mM ethylenediaminetetraacetic acid, and 10 mM dithiothreitol) and sonicated for 5 min (pulse on 2 s, pulse off 3 s, power 180 W), followed by centrifugation at 20,000 × *g* for 30 min. The supernatant was collected and added to dithiothreitol to a final concentration of 10 mM. After incubation in a water bath at 56°C for 1 h, iodoacetamide was quickly added, to a final concentration of 55 mM. After incubation for 1 h in a dark environment, the supernatant solution was supplemented with a fourfold volume of pre-chilled acetone, and then precipitated at −20°C for more than 3 h. After centrifugation at 4°C under 20,000 × *g* for 30 min, the precipitate was dissolved in 400 μL of digestion buffer to a final concentration of 50% triethylammonium bicarbonate (TEAB) and 0.1% sodium dodecyl sulfate (SDS). Subsequently, sonication was performed for 3 min with a pulse on time of 2 s and a pulse off time of 3 s at an ultrasonic power of 180 W, and centrifugation was performed for 30 min at 4°C and 20,000 × *g*. Finally, the supernatant was obtained, and the protein concentration was quantified using the Bradford assay (Supplementary Table 1).

From each sample solution, 100 μg of protein was transferred to a new clean centrifuge tube. The TEAB solution (0.1% SDS) was added to make the protein solution of each sample in the new tube up to the same volume. After adding 3.3 μL of trypsin (1 μg/μL) to the tube for protein digestion, the solution was incubated in a water bath at 37°C for 24 h. Subsequently, the tube was supplemented with 1 μL of trypsin (1 μg/μL), and the mixture in the tube was incubated in a water bath at 37°C for 12 h. After lyophilization of the mixture, 30 μL of TEAB (ddH_2_O:TEAB = 1:1, v/v) was added to the tube to dissolve the peptides. Using an iTRAQ^®^ Reagent-8Plex Multiplex Kit (Applied Biosystems, Foster City, CA, United States), peptides from the eight samples were labeled with the iTRAQ tags as follows: CYR32 (tags 113 and 114), CYR32-5 (tags 115, 116, and 117), and CYR32-61 (tags 118, 119, and 121), respectively (Supplementary Table 1).

The solution containing labeled peptides of each sample was diluted tenfold with buffer A (25% acetonitrile (ACN), 10 mM KH_2_PO_4_, pH 3.0), and the pH was adjusted to 3.0 with phosphoric acid. After centrifugation for 15 min at 15,000 × *g*, the labeled peptides in the supernatant were fractionated using a Phenomenex Luna SCX column (250 mm × 4.60 mm, 100Å) with a high performance liquid chromatography (HPLC) system (Thermo Fisher Scientific, Waltham, MA, United States) at a flow rate of 1 mL/min. The system was equilibrated for 10–20 min with buffer A at a flow rate of 1 mL/min, prior to strong cation exchange fractionation. The HPLC gradient was as follows: 0–45 min, 100% buffer A; 45–46 min, 0–5% buffer B (25% ACN, 2 M KCl, 10 mM of KH_2_PO_4_, pH 3.0); 46–66 min, 5–30% buffer B; 66–71 min, 30–50% buffer B; 71–76 min, 50% buffer B; 76–81 min, 50–100% buffer B; 81–91 min, 100% buffer B. Absorbance was recorded at 214 nm. The eluted peptides were desalted with a Strata-X C18 column (Phenomenex, Torrance, CA, United States), and then dried by vacuum centrifugation at a low temperature.

### Nano LC-MS/MS Analysis

The desalted, dried peptides were resuspended with solvent A (0.1% formic acid (FA) in H_2_O), transferred to an Acclaim PePmap C18-reversed phase column (75 μm × 2 cm, 3 μm, 100 Å, Thermo Scientific), and then separated using a Dionex Ultimate 3000 Nano LC system with a C18 reversed phase column (75 μm × 10 cm, 5 μm, 300 Å, Agela Technologies) at a flow rate of 400 nL/min. The mobile phases were solvent A and solvent B (0.1% FA in ACN). The elution gradient was as follows: 0–10 min, 5% solvent B; 10–40 min, 5–30% solvent B; 40–45 min, 30–60% solvent B; 45–48 min, 60–80% solvent B; 48–55 min, 80% solvent B; 55–58 min, 80–5% solvent B; 58–65 min, 5% solvent B. Subsequently, 16 pre-isolated and purified components of peptides were detected using a Q-Exactive mass spectrometer (Thermo Fisher Scientific, Waltham, MA, United States) with set parameters as follows: polarity, positive ion mode; MS scan range, 350–2000 m/z; MS/MS scan resolution, 17,500; capillary temperature, 320°C; ion source voltage, 1,800 V; MS/MS acquisition modes, higher collision energy dissociation; normalized collision energy, 28.

### Proteomic Data Analysis

The acquired raw mass spectrometry data were processed using the PD (Proteome Discoverer 1.3; Thermo Fisher Scientific, San Jose, CA, United States) software with the following parameters: mass range of parent ion, 350–6,000 Da; minimum number of peaks in MS/MS spectrum, 10; signal-to-noise ratio (S/N) threshold, 1.5. The spectra extracted using the PD software were searched against the Basidiomycota_UniProt database using Mascot 2.3.0 (Matrix Science, London, United Kingdom) with the following identification parameters: fixed modification, carbamidomethyl (C); variable modification, oxidation (M), Gln→Pyro-Glu (N-term Q), iTRAQ 8 plex (K), iTRAQ 8 plex (Y), iTRAQ 8 plex (N-term); peptide tolerance, 15 ppm; MS/MS tolerance, 20 mmu; max missed cleavages, 1; enzyme, trypsin. Based on the Mascot search results and the extracted spectra, protein quantitative analysis was performed using the PD software with the following parameters: protein ratio type, median; minimum peptides, 1; normalization method, median; *P*-value, < 0.05; ratio, ≥ 1.2. In this study, proteins with at least two unique peptides, scores more than 70, *P*-values less than 0.05, and fold changes more than 1.2 or less than 0.83 were identified as DEPs between the different *Pst* strains. The fold changes of the up-regulated DEPs were more than 1.2, and those of the down-regulated DEPs were less than 0.83. For the convenience of expression, CYR32-5 vs. CYR32, CYR32-61 vs. CYR32, or CYR32-61 vs. CYR32-5 was used to represent the combination in which the proteome of the former was compared with that of the latter to screen DEPs.

Functional annotations of the proteins were carried out using the Blast2Go program^1^ (Conesa et al., 2005). The COG annotations of proteins were conducted using WebMGA^2^ (Wu S. T. et al., 2011). The annotations of the identified DEPs at the biological pathway level were performed using the KEGG database^3^. The KOBAS 2.0 software was used to annotate pathways by comparing similar protein sequences (Xie et al., 2011). Signal peptides were predicted and analyzed using the SignalP 5.0 program^4^ (Armenteros et al., 2019).

The mass spectrometry proteomics data have been deposited to the ProteomeXchange Consortium^5^ via the iProX partner repository (Ma et al., 2019) with the dataset identifier PXD018136.

### Relative Quantification of mRNA by qRT-PCR

Using the method described above, 20 mg of the germinated urediospores and germ tubes of each of the three strains CYR32, CYR32-5, and CYR32-61 were collected and treated as a sample. Total RNA from each sample was extracted using the RNeasy kit (Omega) according to the manufacturer’s instructions, and then genomic DNA contaminants were removed by DNase I (RNase-free) treatment. The synthesis of the first-strand cDNA was performed by reverse transcription using the PrimeScript^TM^ RT Master Mix kit (Takara, Dalian, China). Specific primers were designed for the selected DEPs according to their corresponding coding sequences using NCBI Primer-BLAST^6^ (Table 1). *Pst* β-tubulin gene *TUBB* (GenBank accession No. EG374306) was used as the reference gene, and the corresponding primer reported by Huang et al. (2012) (as shown in Table 1) was used in this study. SYBR Green real-time fluorescence quantitative PCR assays were performed using an Applied Biosystems ABI Model 7500 Real Time PCR system. The qRT-PCR reactions were carried out in a total 20 μL volume of reaction mixture containing 10 μL 2 × SYBR Premix DimerEraser, 0.6 μL forward primer (10 μM), 0.6 μL reverse primer (10 μM), 2.0 μL template DNA, 6.4 μL sterile purified water, and 0.4 μL 50 × ROX Reference Dye or Dye II. The amplification procedure was as follows: pre-denaturation at 95°C for 30 s, followed by 40 cycles of 95°C for 5 s, 55°C for 30 s, and 72°C for 30 s. The relative gene expression levels were calculated using the 2^−ΔΔC_T_^ method described by Livak and Schmittgen (2001). The experiment was carried out with three biological replicates.

**TABLE 1:** Sequences of the primers used for real-time fluorescence quantitative PCR assays.

**Accession**	**Forward primer (5′–3′)**	**Reverse primer (5′–3′)**
TUBB	AACAATGTCACAGTGGGCGGTTT	GTGGAAGAGAACATGCTGTCCGT
G9B235	GTCTTCGATTCCCCGGTCAG	CAGTCAAGTATCGGCCGTGT
F4S8X5	TAGCAGGCGAGTCACTAACG	GTCGTCAGTTTCATCGCTGG
V2XWY1	GAACTCTGCAGCTCGACTAT	ATGACCGGACCTGTTACCTT
Q9C1C1	AGGATGGTATGGGCGGTAAAC	GGAGGGCTTCACGTTGTTCA
F4S0Z3	CCGATCACCACCAAGACCAT	AATCTTTCCACCACGAGCGT
F4RWN9	GTTCTCAGAGCCGACGGAAA	CTACTGCATCACCCACGGTT
E3L0W8	GGATACATTGGAGCGGGTGA	CTTCCGATCTGAACGCCCTT
D4QFJ2	GGCCATTTCACAAACGAGGT	ACTCCCACTCGAGCATCT
J6EXB0	CAGAACCAGGGTGAGATGCT	CACCCTCATCGGCGTAAGTA

## Results

### Overview of the Proteomic Data of the Three *Pst* Strains

A total of 337,731 spectra were acquired from the eight samples of the three *Pst* strains, and 52,290 spectra were matched by searching against the Basidiomycota_UniProt database using Mascot 2.3.0 (false discovery rate < 1%). In total, 8,882 peptides and 2,271 proteins were obtained (Supplementary Table 2).

### Acquired DEPs and COG Annotations

**FIGURE 1:**
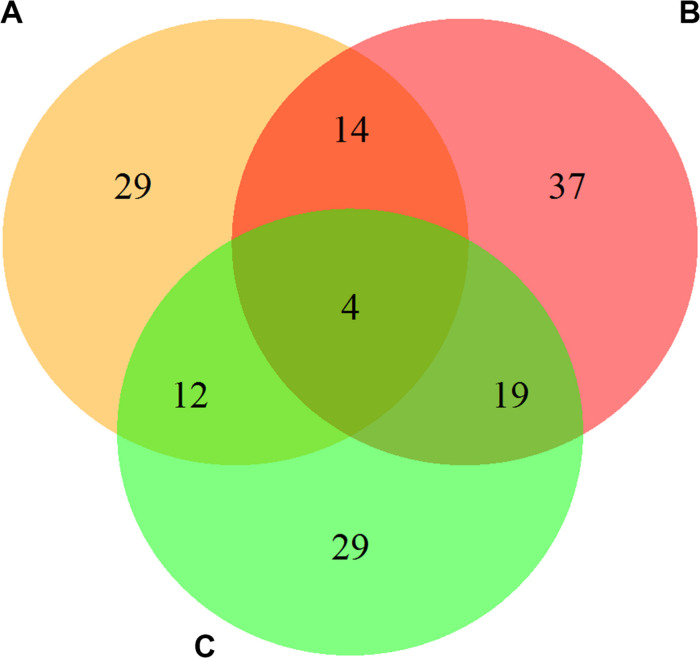
The overlap among the differentially expressed proteins in the germinated *Pst* urediospores in the combinations CYR32-5 vs. CYR32 **(A)**, CYR32-61 vs. CYR32 **(B)**, and CYR32-61 vs. CYR32-5 **(C)** (For each combination, the proteome of the former was compared with that of the latter to screen the differentially expressed proteins).

The results of screening the DEPs in the urediospores and germ tubes after germination in three combinations, CYR32-5 vs. CYR32, CYR32-61 vs. CYR32, and CYR32-61 vs. CYR32-5, are shown in Figure 1. As shown in Figure 1, 59 DEPs were obtained in the combination CYR32-5 vs. CYR32, of which 35 proteins were up-regulated and 24 proteins were down-regulated, and the DEPs E3JUB4 and Q9C1C1 (3.39%) were predicted to contain signal peptides. Seventy-four DEPs were obtained in the combination CYR32-61 vs. CYR32, of which 43 proteins were up-regulated and 31 proteins were down-regulated, and the DEPs Q9C1C1, R7SW21, E3JQF7, E3JUB4, E3JSA3, E3KXP2, and E3K3X8 (9.46%) were predicted to contain signal peptides. Sixty-four DEPs were obtained in the combination CYR32-61 vs. CYR32-5, of which 40 were up-regulated and 24 were down-regulated, and the DEPs E3K302, E3KDD8, E3JSA3, and E3JQF7 (6.25%) were predicted to contain signal peptides. In total, 144 DEPs were obtained in the three comparison combinations, of which four were common in CYR32-5 vs. CYR32, CYR32-61 vs. CYR32, and CYR32-61 vs. CYR32-5. The DEPs that were predicted to contain signal peptides may be secretary proteins or membrane proteins.

**FIGURE 2:**
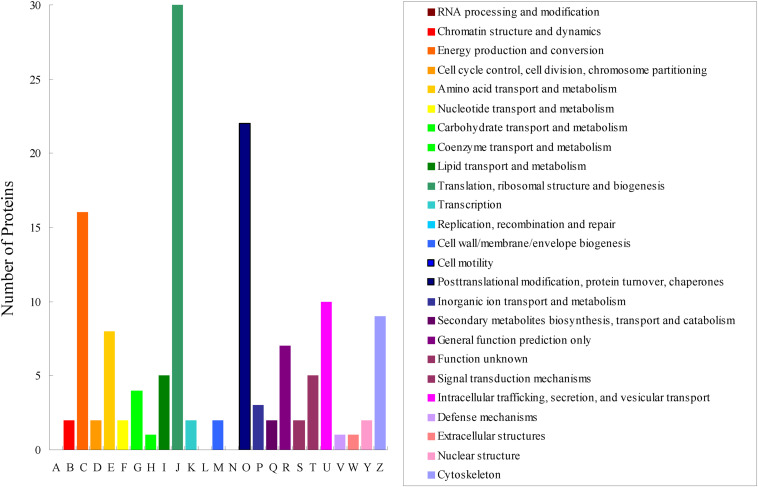
COG functional classification of the differentially expressed proteins acquired via iTRAQ technology in the combinations CYR32-5 vs. CYR32, CYR32-61 vs. CYR32, and CYR32-61 vs. CYR32-5.

All obtained DEPs were subjected to COG functional annotation, and then assigned to 25 categories that were represented by A–Z (Figure 2). Among all the DEPs, seven were annotated with multiple functions, and 22 were not annotated and had no annotation information. The four main functional categories were as follows: [J] translation, ribosomal structure and biogenesis (31, 21.68%), [O] posttranslational modification, protein turnover, chaperones (24, 16.78%), [C] energy production and conversion (17, 11.89%), and [U] intracellular trafficking, secretion, and vesicular transport (11, 7.69%). The detailed functional annotations of the up-regulated DEPs and the down-regulated DEPs are shown in Tables 2, 3, respectively. The acquired DEPs were classified into four major functional categories, including “information storage and processing,” “cellular processes and signaling,” “metabolism,” and “poorly characterized.” Of the up-regulated DEPs, 14, 43, 27, and 4 were involved in “information storage and processing,” “cellular processes and signaling,” “metabolism,” and “poorly characterized,” respectively, and 18 had no annotation information. Of the down-regulated DEPs, 23, 15, 18, and 5 were involved in “information storage and processing,” “cellular processes and signaling,” “metabolism,” and “poorly characterized,” respectively, and 7 had no annotation information.

**TABLE 2:** Functional annotations of the up-regulated differentially expressed proteins acquired in the combinations CYR32-5 vs. CYR32, CYR32-61 vs. CYR32, and CYR32-61 vs. CYR32-5.

**COG functional classification**	**Accession**	**Description**	**Treatment combination**	**Folder change**
**Information storage and processing (14)**				
[B]Chromatin structure and dynamics	E3L109	Histone chaperone ASF	CYR32-61 vs. CYR32-5	1.31
[J]Translation, ribosomal structure and biogenesis	E3K6N0	40S ribosomal protein S0	CYR32-5 vs. CYR32	1.20
	E3KQW3	Eukaryotic translation initiation factor 3 subunit B	CYR32-5 vs. CYR32	1.22
	E6RA36	60S acidic ribosomal protein, putative	CYR32-5 vs. CYR32	1.23
	Q0Z8F6	Elongation factor 1-alpha	CYR32-5 vs. CYR32	1.25
	C0L941	Polyubiquitin-like protein	CYR32-61 vs. CYR32	1.27
	E3KDZ2	Ubiquitin-40S ribosomal protein S27a-2	CYR32-61 vs. CYR32	1.28
	I3VIR3	Elongation factor 1-alpha	CYR32-61 vs. CYR32	1.32
	E2D961	Transcription elongation factor 1 alpha	CYR32-61 vs. CYR32-5	1.31
	E3K5L7	40S ribosomal protein S19-A	CYR32-5 vs. CYR32	1.24
	M7WWX9	60S ribosomal protein l3	CYR32-5 vs. CYR32	1.21
	P51997	60S ribosomal protein L25	CYR32-5 vs. CYR32	1.37
	E3JTG6	Putative uncharacterized protein	CYR32-61 vs. CYR32	1.29
			CYR32-61 vs. CYR32-5	1.21
[K]Transcription	E3L109	Histone chaperone ASF1	CYR32-61 vs. CYR32-5	1.31
**Cellular processes and signaling (43)**				
[U]Intracellular trafficking, secretion, and vesicular transport	F4SA43	Putative uncharacterized protein	CYR32-5 vs. CYR32	1.29
	E3KI07	Putative uncharacterized protein	CYR32-61 vs. CYR32	1.27
	F4RJX3	Putative uncharacterized protein	CYR32-61 vs. CYR32	1.28
	E3L3S6	Putative uncharacterized protein	CYR32-61 vs. CYR32-5	1.22
	L8WPK4	Hsp70-like protein	CYR32-5 vs. CYR32	1.22
			CYR32-61 vs. CYR32	1.21
	F4RWN9	Putative uncharacterized protein	CYR32-5 vs. CYR32	1.42
	E2LWJ3	Uncharacterized protein	CYR32-61 vs. CYR32-5	1.21
			CYR32-61 vs. CYR32	1.33
	E3KQZ6	Putative uncharacterized protein	CYR32-5 vs. CYR32	1.22
[Z]Cytoskeleton	E3KQZ6	Putative uncharacterized protein	CYR32-5 vs. CYR32	1.22
	H6QRQ7	Tubulin beta-1 chain, variant	CYR32-61 vs. CYR32	1.25
	E3KG83	Actin-like protein 2/3 complex subunit 4	CYR32-61 vs. CYR32-5	1.25
	E3KNW6	Profilin	CYR32-61 vs. CYR32-5	1.29
	E3L0W8	Putative uncharacterized protein	CYR32-61 vs. CYR32	1.33
			CYR32-61 vs. CYR32-5	1.70
	J5QLA2	Actin	CYR32-61 vs. CYR32	1.24
			CYR32-61 vs. CYR32-5	1.28
	J9PGK7	Beta-tubulin 1	CYR32-61 vs. CYR32	1.33
			CYR32-61 vs. CYR32-5	1.37
[D]Cell cycle control, cell division, chromosome partitioning	E3KQZ6	Putative uncharacterized protein	CYR32-5 vs. CYR32	1.22
[M] Cell wall/membrane/envelope biogenesis	F4R5Y4	Putative uncharacterized protein	CYR32-5 vs. CYR32	1.25
	G7E8L1	Uncharacterized protein	CYR32-5 vs. CYR32	1.26
			CYR32-61 vs. CYR32	1.25
[O]Posttranslational modification, protein turnover, chaperones	E3JZW9	Putative uncharacterized protein	CYR32-5 vs. CYR32	1.41
	C0L941	Polyubiquitin-like protein	CYR32-61 vs. CYR32	1.27
	E3JS49	Putative uncharacterized protein	CYR32-61 vs. CYR32	1.23
	E3JWN9	Putative uncharacterized protein	CYR32-61 vs. CYR32	1.30
	F4RD53	Ubiquitin carboxyl-terminal hydrolase	CYR32-61 vs. CYR32	1.22
	G7E8A3	Uncharacterized protein	CYR32-61 vs. CYR32	1.21
	I4YJ87	Putative 26S protease regulatory subunit 6B	CYR32-61 vs. CYR32	1.20
	R7SW21	Heat shock protein 70	CYR32-61 vs. CYR32	1.29
	U5H357	Uncharacterized protein	CYR32-61 vs. CYR32	1.27
	E3K302	Putative uncharacterized protein	CYR32-61 vs. CYR32-5	1.23
	E3L494	E3 ubiquitin ligase complex SCF subunit sconC	CYR32-61 vs. CYR32-5	1.21
	L8WPK4	Hsp70-like protein	CYR32-5 vs. CYR32	1.22
			CYR32-61 vs. CYR32	1.21
	Q9C1C1	Cro r II	CYR32-5 vs. CYR32	1.52
			CYR32-61 vs. CYR32	1.35
	E3KTD1	Putative uncharacterized protein	CYR32-61 vs. CYR32-5	1.60
	D8PWL0	Putative uncharacterized protein	CYR32-61 vs. CYR32	1.33
			CYR32-61 vs. CYR32-5	1.21
	E3JQF7	Peptidyl-prolyl cis-trans isomerase	CYR32-61 vs. CYR32	1.21
			CYR32-61 vs. CYR32-5	1.37
	E3KQ21	Peptidyl-prolyl cis-trans isomerase	CYR32-61 vs. CYR32	1.79
			CYR32-61 vs. CYR32-5	1.98
	E3KFZ5	Putative uncharacterized protein	CYR32-5 vs. CYR32	1.40
[T]Signal transduction mechanisms	E3KLJ3	Calmodulin	CYR32-61 vs. CYR32-5	1.35
	F4RVI3	Putative uncharacterized protein	CYR32-61 vs. CYR32-5	1.29
	U5H740	Uncharacterized protein	CYR32-5 vs. CYR32	1.38
	M5FTW6	Calmodulin	CYR32-61 vs. CYR32	1.21
			CYR32-61 vs. CYR32-5	1.35
[Y]Nuclear structure	F4RWN9	Putative uncharacterized protein	CYR32-5 vs. CYR32	1.42
[V]Defense mechanisms	E3KFZ5	Putative uncharacterized protein	CYR32-5 vs. CYR32	1.40
[W]Extracellular structures	E3L2G8	Kinesin family member C1	CYR32-61 vs. CYR32	1.24
**Metabolism (27)**				
[F]Nucleotide transport and metabolism	E3K3Q5	Nucleoside diphosphate kinase	CYR32-61 vs. CYR32	1.27
[G]Carbohydrate transport and metabolism	D4QFJ2	Glyceraldehyde-3-phosphate dehydrogenase	CYR32-61 vs. CYR32	1.27
	F4S9P5	Putative uncharacterized protein	CYR32-61 vs. CYR32	1.34
	F4SBA6	Putative uncharacterized protein	CYR32-61 vs. CYR32-5	1.24
	S7RI63	Transketolase	CYR32-61 vs. CYR32-5	1.26
[I]Lipid transport and metabolism	E3KFZ5	Putative uncharacterized protein	CYR32-5 vs. CYR32	1.40
[C]Energy production and conversion	E3JQW4	Putative uncharacterized protein	CYR32-5 vs. CYR32	1.23
	E3JWK6	Ubiquinol-cytochrome c reductase cytochrome c1 subunit	CYR32-5 vs. CYR32	1.27
	E3K937	Putative uncharacterized protein	CYR32-5 vs. CYR32	1.21
	E3L519	Ubiquinol-cytochrome c reductase iron-sulfur subunit	CYR32-5 vs. CYR32	1.33
	F4S0Z3	ATP synthase subunit beta	CYR32-5 vs. CYR32	1.30
	F4S861	Putative uncharacterized protein	CYR32-5 vs. CYR32	1.26
	F4R4B5	Putative uncharacterized protein	CYR32-5 vs. CYR32	1.33
			CYR32-61 vs. CYR32	1.21
	B0CPP7	Predicted protein	CYR32-5 vs. CYR32	1.41
	M5FPE0	Pyruvate dehydrogenase e1 component alpha subunit	CYR32-5 vs. CYR32	1.27
	A8N2Q4	Inorganic diphosphatase	CYR32-61 vs. CYR32	1.25
			CYR32-61 vs. CYR32-5	1.22
	F4S8X5	Putative uncharacterized protein	CYR32-5 vs. CYR32	1.20
			CYR32-61 vs. CYR32	1.27
			CYR32-61 vs. CYR32-5	1.34
[E]Amino acid transport and metabolism	E3KFZ5	Putative uncharacterized protein	CYR32-5 vs. CYR32	1.40
	S7RH13	Threonine synthase	CYR32-61 vs. CYR32	1.26
	E6R529	Serine hydroxymethyltransferase	CYR32-5 vs. CYR32	1.31
			CYR32-61 vs. CYR32	1.38
	E3JSA3	Pyrroline-5-carboxylate reductase	CYR32-61 vs. CYR32	1.42
			CYR32-61 vs. CYR32-5	1.53
	E3KJR8	Aspartate aminotransferase	CYR32-61 vs. CYR32	1.38
			CYR32-61 vs. CYR32-5	1.23
[P]Inorganic ion transport and metabolism	E3KMT2	Putative uncharacterized protein	CYR32-5 vs. CYR32	1.25
	E3KXP2	Putative uncharacterized protein	CYR32-61 vs. CYR32	1.29
	F4RSD1	Putative uncharacterized protein	CYR32-61 vs. CYR32-5	1.37
[Q]Secondary metabolites biosynthesis, transport and catabolism	E3KWF2	Putative uncharacterized protein	CYR32-61 vs. CYR32-5	1.40
	Q4R0J7	D-arabinitol dehydrogenase 1	CYR32-61 vs. CYR32-5	1.30
**Poorly characterized (4)**				
[R]General function prediction only	F4SA43	Putative uncharacterized protein	CYR32-5 vs. CYR32	1.29
	E3JSL5	Putative uncharacterized protein	CYR32-5 vs. CYR32	1.31
	L8WPK4	Hsp70-like protein	CYR32-5 vs. CYR32	1.22
			CYR32-61 vs. CYR32	1.21
	E3KAU0	Putative uncharacterized protein	CYR32-61 vs. CYR32	1.21
			CYR32-61 vs. CYR32-5	1.26
No annotation (18)	D2SRL3	Translation elongation factor 1-alpha	CYR32-5 vs. CYR32	1.29
	E3KDE9	Putative uncharacterized protein	CYR32-5 vs. CYR32	1.23
	E6RBZ4	Actin-like protein 3 (Actin-related protein 3), putative	CYR32-5 vs. CYR32	1.22
	F4R783	Putative uncharacterized protein	CYR32-5 vs. CYR32	1.25
	E3JXB6	Putative uncharacterized protein	CYR32-61 vs. CYR32	1.25
	E3KKC9	Putative uncharacterized protein	CYR32-61 vs. CYR32	1.21
	E3JWK1	Putative uncharacterized protein	CYR32-61 vs. CYR32-5	1.22
	E3KDD8	Putative uncharacterized protein	CYR32-61 vs. CYR32-5	1.26
	E3KVQ7	Putative uncharacterized protein	CYR32-61 vs. CYR32-5	1.27
	G0SYB3	ATP-dependent RNA helicase dhh1	CYR32-61 vs. CYR32-5	1.23
	K5VLR9	Uncharacterized protein	CYR32-61 vs. CYR32-5	1.30
	M7XSU0	GDP-mannose 4,6-dehydratase	CYR32-61 vs. CYR32-5	1.20
	R7S032	Uncharacterized protein	CYR32-61 vs. CYR32-5	1.35
	E3JUB4	Putative uncharacterized protein	CYR32-5 vs. CYR32	1.25
			CYR32-61 vs. CYR32	1.38
	E3L514	Putative uncharacterized protein	CYR32-61 vs. CYR32-5	1.20
	H6QS83	Putative uncharacterized protein	CYR32-61 vs. CYR32-5	1.46
	E3JQG5	Putative uncharacterized protein	CYR32-61 vs. CYR32	1.34
			CYR32-61 vs. CYR32-5	1.52
	E3K3W4	Glucose-repressible protein	CYR32-61 vs. CYR32	1.80
			CYR32-61 vs. CYR32-5	2.54

**TABLE 3:** Functional annotations of the down-regulated differentially expressed proteins acquired in the combinations CYR32-5 vs. CYR32, CYR32-61 vs. CYR32, and CYR32-61 vs. CYR32-5.

**COG functional classification**	**Accession**	**Description**	**Treatment combination**	**Folder change**
**Information storage and processing (23)**				
[B]Chromatin structure and dynamics	E3JV95	Histone H2B	CYR32-61 vs.CYR32	0.74
			CYR32-61 vs. CYR32-5	0.76
[J]Translation, ribosomal structure and biogenesis	E3KHH0	40S ribosomal protein S22	CYR32-5 vs. CYR32	0.80
	F4RAC5	40S ribosomal protein S0	CYR32-5 vs. CYR32	0.83
	F4RBD4	Putative uncharacterized protein	CYR32-5 vs. CYR32	0.82
	H6QUT2	Glutaminyl-tRNA synthetase	CYR32-5 vs. CYR32	0.82
	M5BM68	Elongation factor 1-alpha	CYR32-5 vs. CYR32	0.82
	B0D8C2	Predicted protein	CYR32-61 vs. CYR32	0.75
	E3KKV5	Large subunit ribosomal protein L14e	CYR32-61 vs. CYR32	0.79
	Q4PS60	Translation elongation factor 1-alpha	CYR32-61 vs. CYR32	0.69
	Q9C1U7	Ribosomal protein L13A	CYR32-61 vs. CYR32	0.78
	E3JVM9	Large subunit ribosomal protein L26e	CYR32-61 vs. CYR32-5	0.81
	F4RJ34	Putative uncharacterized protein	CYR32-61 vs. CYR32-5	0.82
	V5GFY5	60s ribosomal protein L10	CYR32-61 vs. CYR32-5	0.78
	D1MWJ6	Translation elongation factor 1-alpha	CYR32-5 vs. CYR32	0.76
			CYR32-61 vs. CYR32	0.74
	E3L6B3	Eukaryotic translation initiation factor 3 subunit A	CYR32-5 vs. CYR32	0.80
			CYR32-61 vs. CYR32	0.79
	E3K5L7	40S ribosomal protein S19-A	CYR32-61 vs. CYR32-5	0.80
	M7WWX9	60S ribosomal protein l3	CYR32-61 vs. CYR32-5	0.77
	P51997	60S ribosomal protein L25	CYR32-61 vs. CYR32-5	0.57
	E3JQ18	60S ribosomal protein L36	CYR32-61 vs. CYR32	0.75
			CYR32-61 vs. CYR32-5	0.80
	E3KXT5	50S ribosomal protein L22	CYR32-61 vs. CYR32	0.78
			CYR32-61 vs. CYR32-5	0.70
	F4S214	Putative uncharacterized protein	CYR32-61 vs. CYR32	0.83
			CYR32-61 vs. CYR32-5	0.77
	F4SA97	Putative uncharacterized protein	CYR32-5 vs. CYR32	0.73
			CYR32-61 vs. CYR32	0.75
[K]Transcription	E3KH53	Putative uncharacterized protein	CYR32-5 vs. CYR32	0.81
**Cellular processes and signaling (15)**				
[U]Intracellular trafficking, secretion, and vesicular transport	E3KMV4	Putative uncharacterized protein	CYR32-61 vs. CYR32	0.82
	F4S013	Clathrin heavy chain	CYR32-61 vs. CYR32	0.82
	F4RWN9	Putative uncharacterized protein	CYR32-61 vs. CYR32-5	0.79
[Z]Cytoskeleton	K5WMR1	Uncharacterized protein	CYR32-61 vs. CYR32	0.78
	G9B235	Beta-tubulin	CYR32-5 vs. CYR32	0.72
			CYR32-61 vs. CYR32	0.78
[D]Cell cycle control, cell division, chromosome partitioning	E3JWZ6	Cyclin-dependent kinases regulatory subunit	CYR32-61 vs. CYR32	0.83
[O]Posttranslational modification, protein turnover, chaperones	F4RCH7	T-complex protein 1 subunit alpha	CYR32-61 vs. CYR32	0.83
	F4S233	Putative uncharacterized protein	CYR32-61 vs. CYR32-5	0.83
	V2XWY1	Heat shock protein	CYR32-5 vs. CYR32	0.74
			CYR32-61 vs. CYR32	0.68
	E3KTD1	Putative uncharacterized protein	CYR32-5 vs. CYR32	0.77
	M7WQZ1	Heat shock 70kDa protein 1/8	CYR32-61 vs. CYR32	0.70
			CYR32-61 vs. CYR32-5	0.72
[T]Signal transduction mechanisms	E3K0C0	Putative uncharacterized protein	CYR32-61 vs. CYR32	0.75
	U5H740	Uncharacterized protein	CYR32-61 vs. CYR32-5	0.78
[Y]Nuclear structure	E3KMV4	Putative uncharacterized protein	CYR32-61 vs. CYR32	0.82
	F4RWN9	Putative uncharacterized protein	CYR32-61 vs. CYR32-5	0.79
**Metabolism (18)**				
[F]Nucleotide transport and metabolism	F4R3N0	Ribonucleoside-diphosphate reductase	CYR32-61 vs. CYR32	0.78
[H]Coenzyme transport and metabolism	F4RPZ7	Putative uncharacterized protein	CYR32-5 vs. CYR32	0.71
			CYR32-61 vs. CYR32	0.74
[I]Lipid transport and metabolism	E3K845	Acyl-coenzyme A oxidase	CYR32-61 vs. CYR32	0.81
	F4RPF1	Putative uncharacterized protein	CYR32-61 vs. CYR32	0.78
	E3KE31	Putative uncharacterized protein	CYR32-61 vs. CYR32-5	0.80
	E3JUV4	Acyl-CoA dehydrogenase	CYR32-5 vs. CYR32	0.72
			CYR32-61 vs. CYR32	0.82
[C]Energy production and conversion	J6EXB0	ADP, ATP carrier protein 2, (ADP/ATP translocase 2)	CYR32-61 vs. CYR32	0.81
	E3KE31	Putative uncharacterized protein	CYR32-61 vs. CYR32-5	0.80
	E6RF63	ADP, ATP carrier protein 2, mitochondrial (ADP/ATP translocase 2), putative	CYR32-61 vs. CYR32-5	0.79
	B0CPP7	Predicted protein	CYR32-61 vs. CYR32-5	0.66
	M5FPE0	Pyruvate dehydrogenase e1 component alpha subunit	CYR32-61 vs. CYR32-5	0.81
	M5G8Y3	NAD-malate dehydrogenase	CYR32-5 vs. CYR32	0.58
			CYR32-61 vs. CYR32	0.38
			CYR32-61 vs. CYR32-5	0.75
	G7E585	Uncharacterized protein	CYR32-5 vs. CYR32	0.68
			CYR32-61 vs. CYR32	0.47
			CYR32-61 vs. CYR32-5	0.75
[E]Amino acid transport and metabolism	E3KE31	Putative uncharacterized protein	CYR32-61 vs. CYR32-5	0.80
	F4RL47	Putative uncharacterized protein	CYR32-5 vs. CYR32	0.76
	K5V5U7	Uncharacterized protein	CYR32-5 vs. CYR32	0.78
[Q]Secondary metabolites biosynthesis, transport and catabolism	E3KWF2	Putative uncharacterized protein	CYR32-5 vs. CYR32	0.71
	Q4R0J7	D-arabinitol dehydrogenase 1	CYR32-5 vs. CYR32	0.82
**Poorly characterized (5)**				
[S]Function unknown	E3K0C0	Putative uncharacterized protein	CYR32-61 vs. CYR32	0.75
	E3K3X8	Putative uncharacterized protein	CYR32-61 vs. CYR32	0.80
[R]General function prediction only	F4RMJ8	Putative uncharacterized protein	CYR32-61 vs. CYR32-5	0.82
	F4S9E0	Putative uncharacterized protein	CYR32-61 vs. CYR32-5	0.83
	G7E585	Uncharacterized protein	CYR32-5 vs. CYR32	0.68
			CYR32-61 vs. CYR32	0.47
			CYR32-61 vs. CYR32-5	0.75
No annotation (7)	E3JV78	Putative uncharacterized protein	CYR32-61 vs. CYR32	0.83
	E3KR14	Putative uncharacterized protein	CYR32-61 vs. CYR32-5	0.83
	F4RYM8	Putative uncharacterized protein	CYR32-61 vs. CYR32-5	0.81
	A7KKN6	Plasma membrane (H^+^) ATPase	CYR32-5 vs. CYR32	0.81
			CYR32-61 vs. CYR32	0.81
	E3L514	Putative uncharacterized protein	CYR32-5 vs. CYR32	0.81
	H6QS83	Putative uncharacterized protein	CYR32-5 vs. CYR32	0.76
	E3K3W4	Glucose-repressible protein	CYR32-5 vs. CYR32	0.78

### KEGG Pathway Enrichment Analysis of DEPs

**FIGURE 3:**
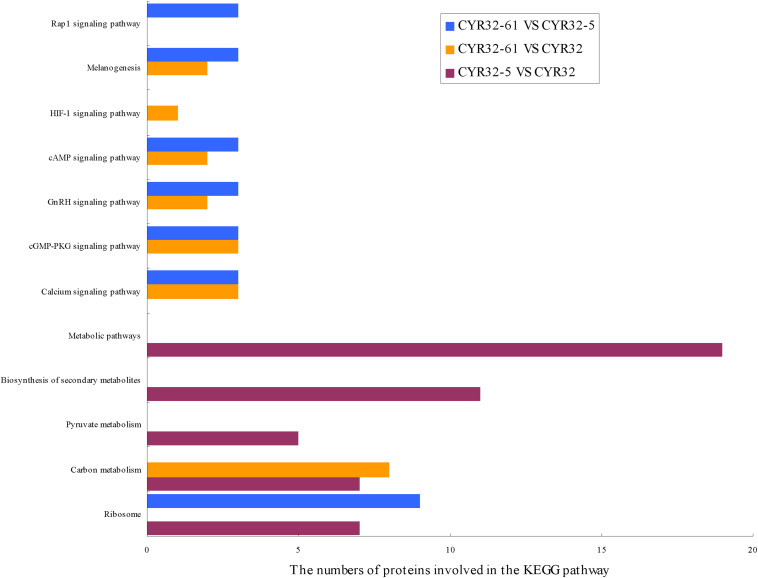
KEGG pathway enrichment of the differentially expressed proteins acquired in the combinations CYR32-5 vs. CYR32, CYR32-61 vs. CYR32, and CYR32-61 vs. CYR32-5 (*P* < 0.05).

The biological pathways that the DEPs were involved in were investigated using the KEGG database, and 5, 7, and 7 significant enriched KEGG pathways (*P* < 0.05) were acquired for the DEPs of CYR32-5 vs. CYR32, CYR32-61 vs. CYR32, and CYR32-61 vs. CYR32-5, respectively (Figure 3 and Supplementary Table 3). The DEPs of CYR32-5 vs. CYR32 were involved in pathways including metabolic pathways, biosynthesis of secondary metabolites, carbon metabolism, ribosome, and pyruvate metabolism. The DEPs of CYR32-61 vs. CYR32 were mainly involved in the carbon metabolism pathway, calcium signaling pathway, and melanogenesis pathway. The DEPs of CYR32-61 vs. CYR32-5 were mainly involved in the ribosome pathway, melanogenesis pathway, and calcium signaling pathway.

### Transcriptional Analysis of DEPs

**FIGURE 4:**
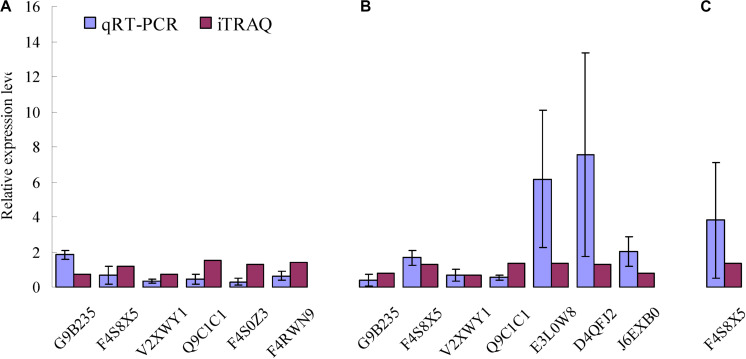
Comparison of encoding gene expression levels and protein levels of the selected enriched proteins identified by iTRAQ analysis in the combinations CYR32-5 vs. CYR32 **(A)**, CYR32-61 vs. CYR32 **(B)**, and CYR32-61 vs. CYR32-5 **(C)**.

To confirm the differences in protein abundance in each of the three combinations including CYR32-5 vs. CYR32, CYR32-61 vs. CYR32, and CYR32-61 vs. CYR32-5, after germination of the urediospores, qRT-PCR was used to investigate the relative expression levels of the genes encoding the DEPs identified by iTRAQ analysis. The relative expression levels of the genes encoding six proteins (G9B235, F4S8X5, V2XWY1, Q9C1C1, F4S0Z3, and F4RWN9) in CYR32-5 vs. CYR32, seven proteins (G9B235, F4S8X5, V2XWY1, Q9C1C1, E3L0W8, D4QFJ2, and J6EXB0) in CYR32-61 vs. CYR32, and one protein (F4S8X5) in CYR32-61 vs. CYR32-5 were determined, and the results are shown in Figure 4. In CYR32-5 vs. CYR32, the relative expression levels of the genes encoding Q9C1C1, G9B235, F4S8X5, F4RWN9, and F4S0Z3 were not consistent with the corresponding protein abundance based on iTRAQ data, but the relative expression of the gene encoding V2XWY1 was consistent with the corresponding protein abundance. In CYR32-61 vs. CYR32, the relative expression levels of the genes encoding Q9C1C1 and J6EXB0 were inconsistent with the corresponding protein levels, and the genes encoding G9B235, F4S8X5, V2XWY1, E3L0W8, and D4QFJ2 were consistent with the corresponding protein levels. In CYR32-61 vs. CYR32-5, the relative expression level of the gene encoding F4S8X5 at the transcriptional level was consistent with the corresponding protein abundance obtained via iTRAQ analysis.

## Discussion

In this study, using the proteomics method based on iTRAQ technology, a total of 144 DEPs were obtained from the germinated urediospores with germ tubes in the CYR32-5 vs. CYR32, CYR32-61 vs. CYR32, and CYR32-61 vs. CYR32-5 conditions. Nine DEPs, including E3JQF7, E3JSA3, E3JUB4, E3K302, E3K3X8, E3KDD8, E3KXP2, Q9C1C1, and R7SW21, were predicted to contain signal peptides. Peptidyl-prolyl *cis-trans* isomerase (PPI, E3JQF7) is a high-efficiency foldase. In biological cells, after ribosome transcription, the peptide chain functions properly only through correct folding and positioning. PPI is associated with the correct folding of a protein (Lu et al., 2007). Heat shock protein 70 (R7SW21) can play an important role in the folding and stretching of newly synthesized polypeptides, can repair denatured proteins, and can prevent protein denaturation by up-regulating protein expression under adverse conditions (Tsan and Gao, 2004). In the present study, both E3JQF7 and R7SW21 were up-regulated in CYR32-61 vs. CYR32, and E3JQF7 was up-regulated in CYR32-61 vs. CYR32-5, indicating that strain CYR32-61 may be more resistant to stress than strains CYR32 and CYR32-5.

The histone chaperone ASF1 (anti-silencing function 1) is a molecular chaperone of histone H3-H4. It has been reported that ASF1 plays important roles in the processes of DNA replication and repair in yeast (Le et al., 1997) and *Arabidopsis* (Zhu et al., 2011). In this study, ASF1 (E3L109) was up-regulated in CYR32-61 vs. CYR32-5, indicating that strain CYR32-61 may be more resistant to stress than strain CYR32-5.

G proteins are involved in various cellular activities, including regulation of intracellular Ca^2+^ concentration, protein synthesis, and the binding of ribosomes to the endoplasmic reticulum (Lagerstedt et al., 2005). The DEPs G9B235, H6QRQ7, and J9PGK7 acquired in this study were involved in G protein regulation. G9B235 was down-regulated in CYR32-5 vs. CYR32, J9PGK7 was up-regulated in CYR32-61 vs. CYR32-5, and H6QRQ7 and J9PGK7 were up-regulated, and G9B235 was down-regulated in CYR32-61 vs. CYR32. Furthermore, the relative expression level of G9B235 determined by qRT-PCR was consistent with the corresponding protein abundance obtained by iTRAQ technology in CYR32-61 vs. CYR32, but the former was inconsistent with the latter in CYR32-5 vs. CYR32. Therefore, the results indicated that there was little correlation between the expression levels at the transcriptional level and the protein level for some DEPs, which was consistent with a previous report by Zhao et al. (2016).

As an important component of the second messenger system, calmodulin plays a key role in the regulation of the calcium signal system, and is associated with physiological metabolism regulation, gene expression, and normal growth and development of cells (Chin and Means, 2000). In both CYR32-61 vs. CYR32 and CYR32-61 vs. CYR32-5, putative uncharacterized protein (E3L0W8) with the role of calcium ion binding and calmodulin (M5FTW6) were up-regulated, and the relative expression level of E3L0W8 determined by qRT-PCR was consistent with the protein level obtained by iTRAQ technology. Calmodulin (E3KLJ3) was up-regulated in CYR32-61 vs. CYR32-5.

**FIGURE 5:**
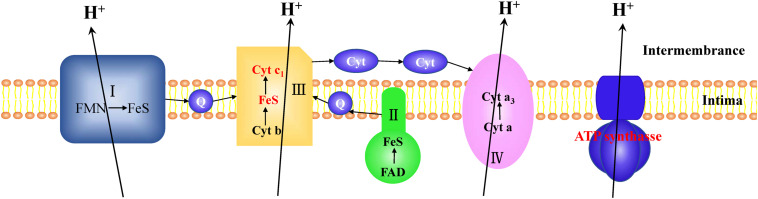
Model for the coupling of electron transport and ATP synthesis. The differentially expressed proteins (marked in red) in the electron transport chain complex III, including ubiquinol-cytochrome c reductase cytochrome c1 subunit (E3JWK6) and ubiquinol-cytochrome c reductase iron-sulfur subunit (E3L519), were up-regulated in CYR32-5 vs. CYR32. The β subunit (F4S0Z3) of ATP synthase marked in red was up-regulated in CYR32-5 vs. CYR32.

In CYR32-5 vs. CYR32, three DEPs involved in energy production, including ubiquinol-cytochrome c reductase cytochrome c1 subunit (E3JWK6), ubiquinol-cytochrome c reductase iron-sulfur subunit (E3L519), and ATP synthase subunit beta (F4S0Z3), were up-regulated. E3JWK6 and E3L519 are components of complex III of the electron transport chain, and are associated with electron transport. F4S0Z3 is the β subunit of the F1 part of ATP synthase, and contains a site to catalyze ATP synthesis. In living organisms, the electron transport chain and ATP synthesis are coupled together (Mitchell, 1961). A model for the coupling of electron transport and ATP synthesis in the combination CYR32-5 vs. CYR32 is shown in Figure 5. In this study, the acquired DEPs involved in the electron transport chain and in ATP synthesis were up-regulated, indicating that the processes of energy production during urediospore germination may be promoted in strain CYR32-5 compared to strain CYR32.

**FIGURE 6:**
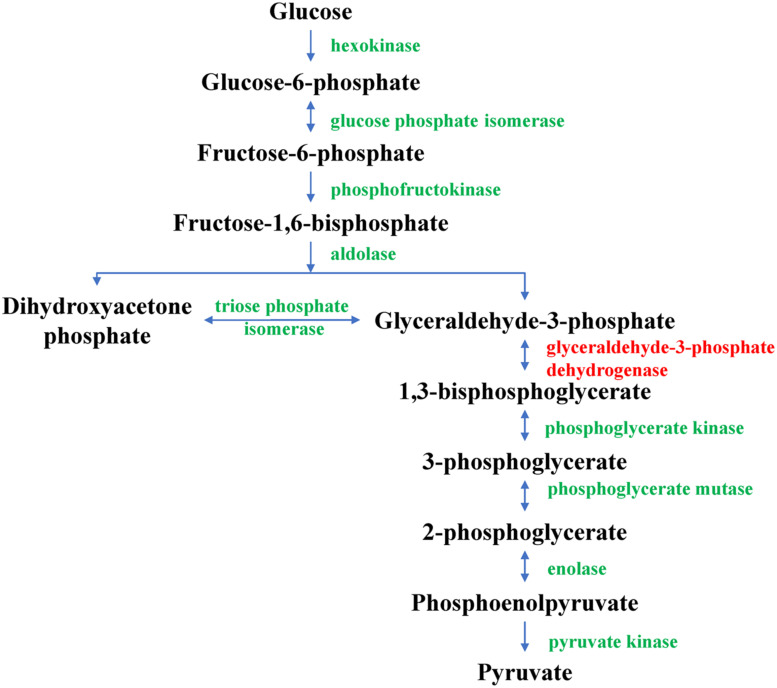
Glycolysis pathway. The glyceraldehyde-3-phosphate dehydrogenase marked in red was found to be up-regulated in CYR32-61 vs. CYR32.

In the glycolysis pathway, glyceraldehyde-3-phosphate dehydrogenase can catalyze the formation of 1,3-diphosphoglycerate from 3-phosphoglyceraldehyde in the presence of NAD^+^ and phosphoric acid; it is, hence, an important enzyme in glycolysis. In this study, D4QFJ2, annotated as glyceraldehyde-3-phosphate dehydrogenase, was up-regulated in CYR32-61 vs. CYR32 according to iTRAQ analysis, which was consistent with the relative expression level determined by qRT-PCR. The results indicated that, during urediospore germination, the glycolysis pathway (as shown in Figure 6) of virulence-mutant strain CYR32-61 may be promoted in comparison with that of strain CYR32. Glycolysis is the main process for the formation of ATP in carbohydrate metabolism. The results obtained in this study showed that the DEPs involved in the glycolysis pathway were up-regulated, further indicating that, during urediospore germination of the virulence-mutant strain CYR32-61 (as compared to the original strain CYR32), polysaccharide may be utilized to form ATP to promote the biological processes of urediospores. In addition, during glycolysis, the final product, pyruvate, can be irreversibly catalyzed by pyruvate dehydrogenase multi-enzyme complex (PDHc) to form acetyl-coenzyme A, which can be oxidatively decomposed in the tricarboxylic acid cycle. Pyruvate dehydrogenase E1 is a component of PDHc, and if its activity is inhibited, aerobic metabolism can be blocked, thus affecting the normal metabolism of living organisms (Danson et al., 1978). In CYR32-5 vs. CYR32, the expression of M5FPE0—annotated as pyruvate dehydrogenase E1 component alpha subunit—was up-regulated, indicating that the formation of ATP during oxidative phosphorylation in the germinated urediospores and germ tubes may be promoted in strain CYR32-5 in comparison with strain CYR32.

Inorganic diphosphatase (A8N2Q4) mainly involved in DNA synthesis, can catalyze the conversion of one molecule of pyrophosphate into two molecules of phosphate, accompanied by the generation of high levels of energy (Kukko and Saarento, 1984). A8N2Q4 was up-regulated in CYR32-61 vs. CYR32 and CYR32-61 vs. CYR32-5 in this study. The results indicated that in comparison with strains CYR32 and CYR32-5, the energy metabolism pathway during urediospore germination may be promoted in strain CYR32-61, which may contribute to the progress in biological processes of urediospores.

It has been reported that UV-B radiation can induce virulence variation in *Pst* (Shang et al., 1994; Huang et al., 2005; Wang et al., 2009; Zhao et al., 2019). Similarly, in studies on two other important pathogens—*Puccinia triticina* (Neugebauer et al., 2018) and *P*. *graminis* f. sp. *tritici* (Zhang et al., 2017), causing wheat leaf rust and wheat stem rust, respectively, it has also been reported that mutation may be the main mechanism of virulence variation of the two pathogens, thus resulting in the loss of wheat resistance and severe losses of wheat yield. In this study, after the germination of urediospores, the expression levels of many proteins in UV-B-induced virulence-mutant strains, CYR32-5 and CYR32-61, were different from those in CYR32, the original strain. The changes in the related biological processes in which the DEPs are involved may result in changes in virulence phenotypes of *Pst* and may be responsible for virulence variation. To further explore the virulence variation mechanisms of *Pst*, it is of great importance to conduct studies by pathogen sequencing at the genome level and to investigate the metabolomics of *Pst* after mutations.

## Conclusion

In this study, using the proteomics method based on iTRAQ technology, a quantitative proteomic analysis of the germinated urediospores (with germ tubes) of the original strain, CYR32, and two UV-B-induced virulence-mutant strains, CYR32-5 and CYR32-61, was undertaken. A total of 2,271 proteins were identified in eight samples of the three *Pst* strains, and 59, 74, and 64 DEPs were obtained in each of the three combinations, i.e., CYR32-5 vs. CYR32, CYR32-61 vs. CYR32, and CYR32-61 vs. CYR32-5, respectively. The identified DEPs were mainly involved in energy metabolism, carbon metabolism and cellular substance synthesis. The relative expression levels of the genes encoding some DEPs, quantified using qRT-PCR, were consistent with the corresponding protein abundance obtained via iTRAQ analysis. Compared with the original strain, CYR32, the DEPs involved in stress-related and energy metabolism were up-regulated in the virulence-mutant strains, indicating that the virulence-mutant strains, CYR32-5 and CYR32-61, were more tolerant to stress than the original strain. These results are of great significance for further studies on the mechanisms of *Pst* virulence variation and for implementing control measures for wheat stripe rust.

## Data Availability Statement

The datasets generated for this study can be found in the ProteomeXchange Consortium (http://proteomecentral.proteomexchange.org) via the iProX partner repository with the dataset identifier PXD018136.

## Author Contributions

HW contributed conception of the study. HW and YaZ designed the experiments. YaZ, PC, and YuZ performed the experiments. YaZ and HW analyzed the data and wrote the draft of the manuscript. All authors contributed to manuscript revision, read and approved the final version of the manuscript.

## Conflict of Interest

The authors declare that the research was conducted in the absence of any commercial or financial relationships that could be construed as a potential conflict of interest.

## Footnotes

1 https://www.blast2go.com/

2 http://weizhong-lab.ucsd.edu/metagenomic-analysis/server/cog/

3 http://www.genome.jp/kegg/

4 http://www.cbs.dtu.dk/services/SignalP/

5 http://proteomecentral.proteomexchange.org

6 https://www.ncbi.nlm.nih.gov/tools/primer-blast/
